# Food Insecurity across Age Groups in the United States during the COVID-19 Pandemic

**DOI:** 10.3390/ijerph21081078

**Published:** 2024-08-16

**Authors:** Zhongqi Fan, Amy M. Yang, Marcus Lehr, Ana B. Ronan, Ryan B. Simpson, Kimberly H. Nguyen, Elena N. Naumova, Naglaa H. El-Abbadi

**Affiliations:** 1 Friedman School of Nutrition Science and Policy, Tufts University, Boston, MA 02111, USA; 2 Jean Mayer USDA Human Nutrition Research Center on Aging, Tufts University, Boston, MA 02111, USA; 3 Rollins School of Public Health, Emory University, Atlanta, GA 30322, USA

**Keywords:** food insecurity, food insufficiency, older adults, COVID-19 pandemic, Household Pulse Survey

## Abstract

Food insecurity increased during the COVID-19 pandemic, but the impact varied across different age groups during the prolonged public health emergency. This study sought to describe national food insecurity prevalence by adult age group at multiple stages of the pandemic and explore differences by demographic characteristics. Data were from the nationally representative US Census Bureau’s Household Pulse Survey from April 2020 to May 2023 (N = 4,153,462). Locally weighted scatterplot smoothing (LOESS) regression analysis identified change points in food insecurity trends, segmenting the timeline into three periods: (1) April 2020–March 2021, (2) April 2021–May 2022, and (3) June 2022–May 2023. Logistic regression models examined associations between age, time period, and self-reported household food insecurity; covariates included demographics, socioeconomic status, household structure, and food support program usage. Overall, 9.3% of respondents experienced food insecurity, ranging from 3.5% among those aged ≥75 to 12.2% for ages 35–44 years. Significant interaction between age group and time period indicated inconsistency in the age-food insecurity association during the pandemic (*p* < 0.001). From Period 1 to 3, the proportion of food-insecure adults aged ≥65 rose from 9.2% to 13.9%. Across all age groups, higher odds of food insecurity were found among Black, Hispanic/Latino, or Other/Multiracial respondents; those with less than a Bachelor’s degree; those with incomes below USD 35,000; those unemployed for reasons other than retirement; and non-homeowners (*p* < 0.001). The results show that trends and characteristics associated with food insecurity varied across age groups and time periods. Continuous monitoring of food insecurity during emergencies is critical to identify vulnerable populations and timely interventions.

## 1. Introduction

The COVID-19 pandemic exposed weaknesses in food production systems [1], affecting food availability and prices [2], which, along with economic fluctuations and increased unemployment [3,4], sharply increased food insecurity rates across the United States [5]. In addition to magnifying pre-existing food insecurity among low-income households that were already struggling to meet their needs [6], many households experienced food insecurity for the first time [7]. It has been established that food and nutrition disparities across certain population groups in the U.S. were exacerbated after the start of the pandemic [8,9].

Age is a prominent demographic factor for risks and harms related to insufficient food access. Older adults (65+) face unique challenges, such as an increased risk of adverse COVID-19 outcomes, mobility limitations [10,11], and fixed incomes [12], which may elevate their risk of food insecurity during prolonged emergencies. Food insecurity among older adults is an issue of public health concern, as insufficient or unhealthy dietary intake underlies and exacerbates many disease and disability conditions associated with aging. Older adults who are food-insecure have limited daily activities compared to those who are food-secure [13], have a higher risk of suffering from both physical and mental health problems [14], and are more likely to be hospitalized [15].

Pre-pandemic, in 2019, approximately 7.2% of US adults age 65 and older were food-insecure, equating to 5.3 million older Americans [16]. However, previous studies indicate a decrease in food insecurity prevalence with increasing age [17,18,19]. While most of these studies were conducted using a single or limited cross-sectional view of the US population, the COVID-19 pandemic officially lasted for over 3 years, based on the US public health emergency designation, from 31 January 2020 until 11 May 2023 [20]. This study therefore aims to delve deeper into the trends of food insecurity by age group during this period.

This study aims to characterize the prevalence of food insecurity across age groups during the pandemic, identify significant changes in food insecurity trends, and evaluate the factors influencing these trends. 

## 2. Materials and Methods

### 2.1. Data and Sample

Data was obtained from the Household Pulse Survey (HPS), conducted by the US Census Bureau. The objective of the HPS was to measure the effects of the COVID-19 pandemic on households across the United States from a social and economic perspective, and to evaluate how people respond to emergencies. The HPS is a nationally representative survey comprised of rapid response questionnaires, administered as a 20-min survey using a Qualtrics platform to collect data systematically and continuously from the US population in approximately 2-week intervals. The HPS implementation uses a multi-level sampling design that provides a representative sample of responses for all US states and 15 Metropolitan Statistical Areas (MSAs) [21]. In the data collection, the Census Bureau’s Master Address File (MAF), with approximately 140,000,000 valid housing units, was used as the source of sampled housing units [22]. The HPS used a rapid deployment internet and telephone interview system, in which email and mobile telephone numbers from the Census Bureau Contact Frame were paired with addresses in the MAF. The selected households were contacted by email and/or text, depending on the information available.

Declaration of COVID-19 as a public health emergency started 31 January 2020 and spanned until 11 May 2023. The HPS implementation began 23 April 2020, with surveys collected approximately every two weeks. All available weeks of data from the HPS during the national public health emergency time period were included in this study, using microdata files from survey weeks 1 through 57. These were obtained from the HPS Public Use Files webpage (https://www.census.gov/programs-surveys/household-pulse-survey/datasets.html, accessed on 19 October 2022), representing a time period starting with the first survey available, on 23 April 2020, through 8 May 2023, which corresponded with the end of the Federal Public Health Emergency Declaration (week 1: 23 April–5 May 2020; week 57: 26 April–8 May 2023).

### 2.2. Inclusion and Exclusion Criteria

All participants who were aged 25 years and older and provided a response to the current food sufficiency question were eligible for analysis. Participants aged 18–24 were excluded due to the high likelihood of being in school full-time, as well as having a different set of influences on food security than adults who were older, such as having lower paying jobs, less stable jobs, and less independence [23]. Participants with missing responses (i.e., question seen but skipped, or missing) were excluded from analysis.

### 2.3. Measures

#### 2.3.1. Dependent Variable: Household Food Insecurity

The primary outcome variable of this study was self-reported food insecurity from the survey question of current food sufficiency: “In the last 7 days, which of these statements best describes the food eaten in your household?” The HPS’s food insufficiency question is one of two questions the HPS adopted from the Household Food Security Survey Module (HFSSM) used for the ERS Food Security Supplement report for the annual assessment of national prevalence of household food insecurity. The HFSSM is considered the standard reference measure in the US, comprising 10 questions (or 18 questions for households with children) to reflect household food access and intake during the designated calendar year [24,25]. The HPS’s use of this question on food sufficiency for the prior 7 days is intended to capture rapid shifts in food security status. We converted this to a binary outcome variable, and categorized respondents as either *food secure* if they chose responses 1 or 2: “Enough of the kinds of food (I/we) wanted to eat” or “Enough, but not always the kinds of food (I/we) wanted to eat”; *food insecure* if they chose responses 3 or 4: “Sometimes not enough to eat” or ”Often not enough to eat”. Responses classified as “Question seen but category not selected (−99)” or ”Missing/Did not report (−88)” were not included in the analysis.

#### 2.3.2. Focal Independent Variable: Age

Continuous age (years) was approximated by the year of the survey response minus the reported year of birth, with participants aged from 25 through 88 years included. Participants were then grouped by decade of age: 25–34, 35–44, 45–54, 55–64, 65–74, 75 and above years. Age group 45–54 was used as the reference group in regression analysis.

#### 2.3.3. Regression Covariates

We controlled for factors that may influence the association between age and food insecurity, including demographic, household structure, and socioeconomic status, as described below, with (*) to indicate the reference group.

Demographic variables included sex, race/ethnicity group, and region of the US. Sex was categorized as Male* and Female. Categories for the combined race and ethnicity variable were non-Hispanic White*, non-Hispanic Black, Hispanic/Latino, Asian, and Other/Multiple. Regions in the US were categorized as Northeast* (CT, ME, MA, NH, RI, VT, NJ, NY, PA), Midwest (IL, IN, MI, OH, WI, MN, IA, KS, MO, NE, ND, SD), South (DE, DC, MD, VA, WV, FL, GA, NC, SC, AL, KY, MS, TN, AR, LA, OK, TX), and West (NM, CO, MT, UT, WY, AZ, NV, ID, CA, HI, AK, OR, WA).

Household structure variables included marital status, number of people in the household, and the presence of children in the household. Marital status was categorized as Married*, Widowed/Divorced/Separated, and Never married. Household structure was categorized as 1 adult and no children <18 years of age, 2+ adults and no children <18 years of age*, 1 adult and any children <18 years of age, and 2+ adults and any children <18 years of age.

Socioeconomic status variables included total household annual income level, education level, employment status, and housing tenure. Household income was converted from an eight-category response to four categories: <USD 35,000, USD 35,000 to <USD 75,000*, USD 75,000 to <USD 150,000, and USD 150,000 and above. Education level was re-categorized as high school graduate or less, some college or an Associate’s degree; Bachelor’s degree*, and graduate degree and above. Employment status was categorized as currently working*, retired, not working and involuntarily unemployed, not working and personal illness or caregiver, not working and unknown or other reason. Housing tenure was categorized as owned*, mortgage or loan, rented, and occupied without pay. 

#### 2.3.4. Food Support and Spending

The HPS did not include questions involving receipt of Supplemental Nutrition Assistance Program (SNAP) support in the survey until week 13 (19–31 August 2020). The questions regarding SNAP varied at different survey weeks and included free food, binary SNAP receipt, and month receiving SNAP support. Therefore, all forms of SNAP receipt were harmonized into a single binary variable for a given survey week and included in the descriptive analysis only. Receipt of free food within the prior 7 days, in the form of groceries or meals, was captured as a binary variable (yes/no). Household money spent within the last 7 days on food to be prepared and eaten at home, or prepared outside the home, were recorded as amount between USD 0–900 or USD 0–500, respectively. The HPS did not include questions on household spending in weeks 35–45. Thus, like the SNAP data, these were only used in the descriptive analysis.

#### 2.3.5. Variable Adjustment

Certain HPS survey questions were modified over time to capture developing and critical information over the course of the pandemic on topics such as food insecurity and SNAP receipt. Such variables were therefore re-leveled, with answer choices combined in order to optimize continuity over all survey weeks. Also, some variables were not present for the full range of the survey, weeks 1–57, as certain variables were added or dropped. Some analysis therefore only covers time periods specific to variable availability and consistency. Variables not available for the full survey, weeks 1–57, are noted, with their availability indicated in Supplementary Table S1. 

#### 2.3.6. Imputations and Missingness

There were some modifications to the dataset prior to public release, in which age, sex, race/ethnicity, educational attainment, number of adults, and number of children in the household were imputed for the respondents who failed to provide this information, using simple hot-deck imputation [26]. However, missing data were still present for certain variables, potentially due to respondents refusing to answer questions on sensitive issues or failing to understand the question, or errors incurred during the data collection and cleaning. There were two types of missing data in this dataset, “Question seen but category not selected”, coded as −99 in the dataset, and “Missing/Did not report”, coded as −88. These two types of missingness were combined for an overall assessment of missingness in the research analysis. The percentage of missingness was evaluated for all variables prior to analysis: food insecurity (10.4%), marital status (0.91%), income level (23.4%), employment status (1.93%), housing tenure (19.25%), SNAP use (19%), received free groceries or meals (11%), money spent on groceries in the last week (17%), and money spent on prepared meals in the last week (18%) (corresponding to variables with missing responses in Table 1 and Table S1).

### 2.4. Analytic Plan

#### 2.4.1. Data Aggregation and Weighting

Survey weeks 1–57 were aggregated into a single data set representing approximately 3 years of survey data (23 April 2020–8 May 2023) for a total sample size of N = 4,261,633. Person weights and replicated person weights were divided by 57 to maintain the population size estimate in the aggregated dataset. The resulting population estimate was the average of the population estimates for weeks 1–57 (N = 229,352,105). All subsequent estimates and models were built using the *survey* R package. The 80 replicate weights were used for variance estimation using Successive Difference Replication (SDR), as specified by the Census Bureau.

#### 2.4.2. Aggregate Cross-Sectional Time

The initial food security analysis was conducted across the entire dataset, without distinction of time (Table 1). Individuals under 25 were removed for analysis for a sample size of N = 3,846,132. Overall proportions were calculated within each demographic characteristic, along with associated proportions for food-secure and food-insecure individuals. Missing values were included but are not shown in the percentages displayed in Table 1. Second order Rao-Scott chi-square tests were used to test for differences in demographic distributions between food-secure and food-insecure groups (missing values excluded).

#### 2.4.3. Time-Period Analysis

The number of days covered by each survey ranged from 4 to 14, and averaged 11 (mode 12 days), although each survey was administered typically over 5 days from 23 April to 21 July 2020 (weeks 1–12), and thereafter typically over 12 days from 19 August 2020 to 8 May 2023 (weeks 13–57). Days between surveys ranged from 2 to 51 and averaged 9, with the frequency diminishing after survey week 39 ended (corresponding to 11 October 2021). An average of 4 days passed between survey weeks 1–39 (mode 2 days), while the average for days between survey weeks for weeks 40–57 was 20 (mode 16 days).

Due to this variability in survey weeks, we converted survey week start dates to days from HPS initiation on 23 April 2020, resulting in a start date variable ranging from days 0–1098. The weight-adjusted food insecurity prevalence was then calculated at each timepoint using the *survey* package in R [27]. Given the nonlinear nature of potential temporal fluctuations [28], locally estimated scatterplot smoothing (LOESS) was used to estimate the food insecurity trend over time. Inflection time points were used to determine when the maximum (peaks) or minimum (valleys) food insecurity proportions of the population occurred. Time periods were then delineated by the midpoints between these peaks and valleys.

#### 2.4.4. Summary Statistics

We first examined and compared the prevalence of food insecurity across all covariates overall, along with some variables in the context of age group. We further examined the use of food program assistance in the study population, and assessed the prevalence of food insecurity among participants who reported that they used SNAP or received free groceries or meals. The chi-square test was applied to assess significant differences in food program use proportions, and the median money spent on food was reported as interquartile ranges.

The analysis was repeated across identified time periods to compare differences in population groups during different periods of the pandemic (Table 2).

#### 2.4.5. Logistic Regression Modeling

To assess the association between food insecurity and age, a logistic regression model was constructed as a function of age group by decade, with consideration of changes over time through the inclusion of an interaction term between age groups and time periods (Model 1). Multivariable logistic regression models were constructed to estimate the odds of food insecurity as a function of age and time period, adjusted for demographic, socioeconomic, and household structure variables. These were built as demographic variables (Model 2), with additional socioeconomic status and household structure variables (Model 3), each including an assessment of the interaction between age and time. Adjusted odds ratios with 95% confidence intervals were estimated to assess the strength of the association of outcomes with covariates, with the significance level set at *p* < 0.05.

#### 2.4.6. Software

R version 4.3 was used to prepare the dataset and for statistical analysis. Weighted proportions, regressions, and statistical tests were performed with the R package *survey* version 4.4. 

## 3. Results

A total of 4,283,916 individuals aged 18 and older responded to HPS surveys weeks 1–57, all of whom were eligible to provide information related to food security status. Respondents under age 25 were excluded, leaving 4,153,462 for the remaining analysis, representative of 229,352,105 US adults aged ≥25 years after weight adjustment.

### 3.1. Characteristics of Population Food Security Status and Aggregate Time

**Figure 1:**
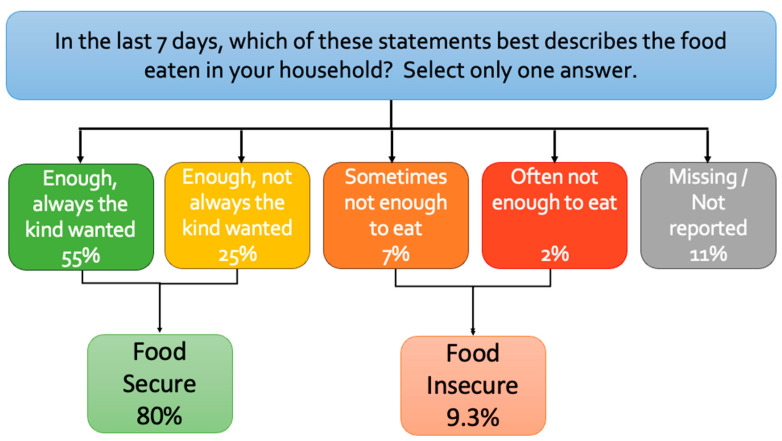
Household food sufficiency for the last 7 days.

Figure 1 shows the distribution of responses to the question of household food sufficiency over the prior seven days. Overall, 9.3% of this population reported being food-insecure (7.2% said they sometimes, and 2.1% said often did not have a sufficient quantity of food), 80.3% were food-secure (55.4% indicated they had enough food, and of the kinds they wanted to eat; 24.9% said they had enough food, but not always of the desired kind), and 10.4% had missing responses for food security.

Table 1 presents weighted summary statistics for the sample population’s demographic, socioeconomic, and household structure characteristics in total, as well as by food security status.

*Among demographic variables*: The age distribution in the population was roughly one quarter (23.7%) aged 65 years and older, and three quarters aged 25–64 years. The food insecurity prevalence decreased with increased age, ranging from roughly 25% for those aged 44 years and younger, down to 2.6% for those aged 75 years and older (*p* < 0.001). Almost two-thirds of participants were non-Hispanic Whites (63%), but they were less than half (44.3%) of those reporting food insecurity. Asian was the only minority group to have a lower proportion in the food-insecure group than their presence in the population (3% vs. 5.3%, respectively). Meanwhile, non-Hispanic Black respondents were one fifth of those who were food-insecure, and Hispanic/Latino were one quarter, despite being only 11.7% and 16.4% of the population, respectively. Almost a third of individuals had either a Bachelor’s or graduate degree, while they accounted for only 10.5% of those food-insecure; the remaining nearly 90% of food-insecure respondents had completed less than a four-year college degree.

*Among socioeconomic variables*: Of those who provided their household income and reported being food-insecure, 75% earned less than USD 75,000, and 50% less than USD 35,000. However, 23.4% of household incomes were missing data, the majority of which were from the food-insecure group. More than half (56%) of the respondents were currently working. Of those not working, 18% were retired, and 24% were unemployed either involuntarily, due to personal illness or having a caregiver role, or for an unspecified reason. Among food-insecure respondents, 42.4% were currently employed, only 7% were retired, and any other reason for not working had a two to three times higher representation among the food insecure than their proportion in the overall population. For housing tenure, approximately 20% of all respondents owned their home outright, while 37% had a home mortgage or loan. Approximately 22% of respondents rented, and 1.3% occupied a home without payment. Nearly 20% of the surveys were missing housing tenure responses. Almost half (46.1%) of those reporting food insecurity rented their homes, as opposed to owning outright (11.7%) or having a mortgage/loan (24.3%). For region of residence, nearly 40% of the respondents resided in the South, with 23.6% in the West, 20.6% in the Midwest, and 17.4% in the Northeast, and these proportions varied little by food security status.

**Figure 2:**
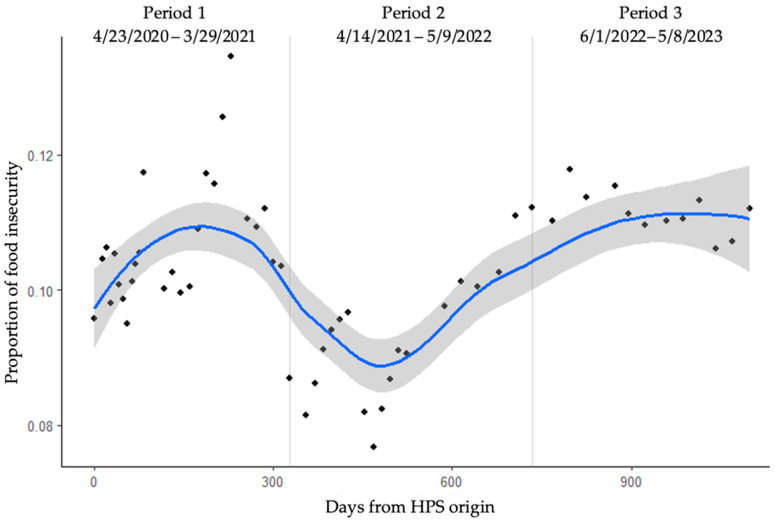
Population estimates for food insecurity. Data were fitted with a LOESS regression (blue) and time periods were defined as the midpoints between the minimum and maximum peaks of the regression curve. Shaded region represents the 95% CI of the regression.

LOESS regression analysis was used to identify timepoints of the highest and lowest proportions of food insecurity (Figure 2). Peaks were found on day 174 (Week 17) and day 986 (Week 53), while the minimum between them was on day 482 (Week 36). The midpoints between were used to determine time periods of analysis. Midpoint 1 was set to day 328 (Week 27) and 734 (Week 45). The time periods were therefore designated as Period 1: Weeks 1–27, corresponding to dates 23 April 2020–29 March 2021, and containing the first maximum; Period 2: Weeks 28–45, corresponding to dates 14 April 2021–9 May 2022, which contained the minimum; and Period 3: Weeks 46–57, corresponding to dates 1 June 2022–8 May 2023, which contained the next highest prevalence of food insecurity.

**Figure 3:**
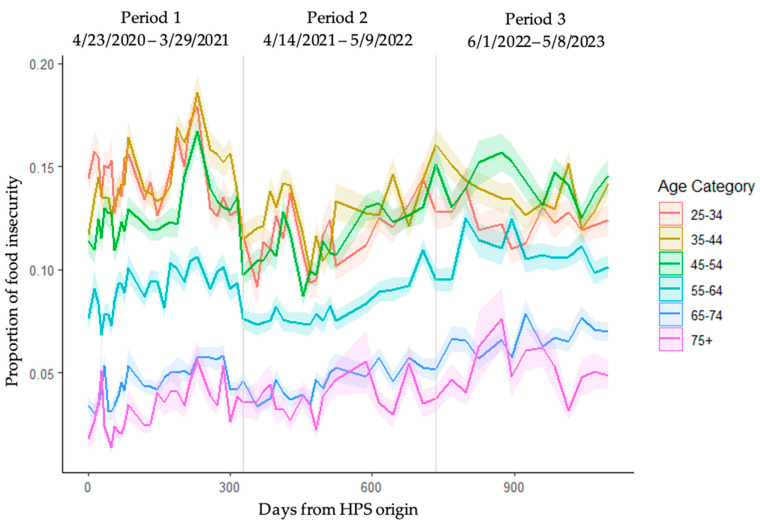
Population estimates for food insecurity by age group. Estimates were calculated as survey means; shaded regions represent standard error.

Food insecurity prevalence by age group was plotted over the course of time (Figure 3). For the six age groups, the pattern of food insecurity prevalence in those aged 25–54 years old seemed highly aligned among the groups, and those aged 65 and above similarly aligned between the groups, with ages 55–64 somewhat between the younger versus older age groups. In the younger age group, food insecurity was mostly stable through ~Day 150, and then rose quickly, while in the ≥65 group, there was an immediate rise in food insecurity, but overall, it was not as steep as in the younger group. Older age did not exhibit great fluctuations in food insecurity compared to those of younger ages.

### 3.2. Characteristics of Population Food Security Status with Time-Period Analysis

Demographic and socioeconomic variables in relation to food security status were further evaluated across the three identified time periods, 1, 2, and 3, with the findings shown in Table 2. The change in the food insecurity proportion from Period 1 to Period 3 were calculated, and values greater than 10% of the Period 1 prevalence were considered to be meaningfully different for either an increase or decrease. These results showed that younger age groups (age 25–44) experienced higher food insecurity in the first time period of the pandemic compared to the third, while age groups 45 years and above had an increase between Periods 1 and 3. There was also an increase in food insecurity over time both among respondents who were working at the time as well as those who were retired, or reported not working without giving a reason. However, involuntarily unemployed respondents had a lower prevalence of food insecurity by Period 3. The food insecurity prevalence increased in single adult households with no children, as well as among respondents who owned their home. Meanwhile, non-Hispanic Black respondents reported a lower proportion of food insecurity in Period 3 compared to Period 1, while no other race/ethnic group had a substantial shift in proportions. The proportion of food-insecure respondents not using SNAP benefits also decreased.

The prevalence of food insecurity did not meaningfully change (>10% of baseline) between time periods for respondents by sex, education level, income level, marital status, or region of residency in the US (Table 2).

### 3.3. Food Support Usage and Food Spending by Aggregate Time

Supplementary Table S1 shows the use of food support programs and food spending. The results indicate that approximately 7% of respondents received free groceries or meals over the preceding week, with 11% missing responses. Also, 11% of the population reporting use of SNAP for the time period captured (Weeks 13–57), although with a near 20% missingness for this question. Food insecurity was present whether or not respondents used SNAP or received free groceries or meals but was at significantly higher proportions among those who did—rising to 26.5% among SNAP-users, and 26.1% for those receiving free food, versus 7.9% and 8.9% who reported food insecurity among those who did not receive SNAP or free food, respectively (*p* < 0.001 for both). Among respondents who were food-insecure, 32% indicated using SNAP benefits, and 19% received free groceries or meals.

Responses regarding money spent over the prior 7 days on food at home (possible range USD 0–900) or on prepared meals (possible range USD 0–500), the median and interquartile range were reported due to skewed distributions. These values were available for Weeks 1–34 and 46–57. Respondents reported spending a median of USD 200 (IQR USD 100–300) on food prepared/eaten at home, and these values were the same for food-secure and food-insecure respondents. However, because household size influenced the amount of spending on food purchases, and food-insecure households more often had more members, the per capita spending was also calculated. These were USD 66.70 (IQR USD 42–100) for food-secure households, versus USD 50 (IQR USD 27–100) per member of food-insecure households (Supplementary Table S1). For prepared meals eaten out, the median amount spent was USD 50 (IQR USD 20–100) in total, the same as for those who were food-secure, but reduced to USD 40 (IQR USD 0–120) for those who were food-insecure. By per household member, this shifted to median USD 20 (IQR USD 7–40) for food-secure households, and USD 10 (IQR USD 0–38) for food-insecure households.

### 3.4. Logistic Regression Models

Table 3 shows the results of the three logistic regression models. Model 1 assessed the odds of food insecurity as a function of age group, time period, and the interaction between them. Relative to ages 45–54 years, those younger showed slightly but significantly higher food insecurity, while older respondents showed an increasingly larger reduction in the odds of food insecurity, controlling for time period. The time period of the pandemic also had an effect on food security status, indicating that Period 2 (April 2021–May 2022) had slightly lowered food insecurity than the initial year of COVID-19 (OR 0.91, 95% CI 0.87–0.95). However, the third Period of the pandemic (June 2022–May 2023) showed that the odds of food insecurity increased significantly (OR 1.15, 95% CI 1.10–1.20) when holding age constant. There were significant interactions between some age groups and time period, indicating the association between age and food security status was not consistent for all age groups over the course of the pandemic.

Model 2 included additional assessments of sex, race/ethnicity, and region of residence in the US (Table 3). The association between age and food insecurity remained consistent with Model 1 after controlling for these variables, as did time period and the interaction between them. Women tended to be more food-insecure than men (OR 1.14, 95% CI 1.09–1.19). Additional information on differences in the proportion of food insecurity by population characteristic for men and women can be found in Supplementary Table S2.

**Figure 4:**
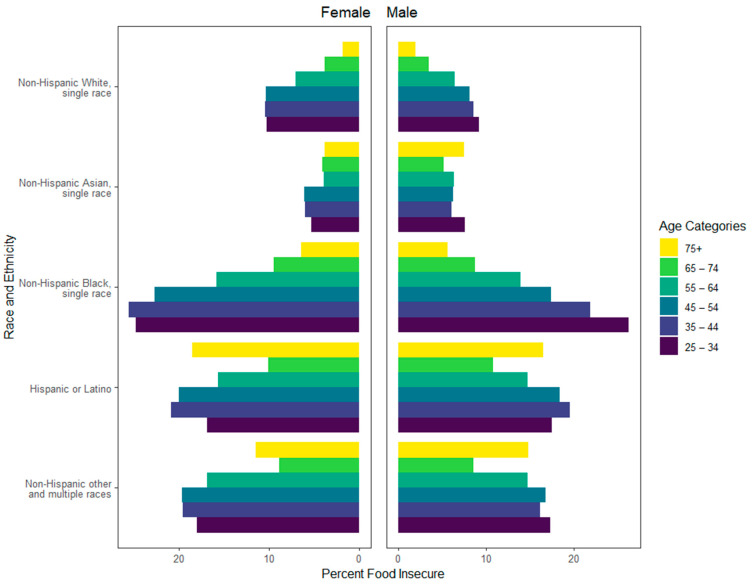
Food insecurity by age, sex, and race/ethnicity. Bars represent the percentage of food insecurity within each subgroup.

Compared to non-Hispanic White respondents, the odds of food insecurity estimates were more than double for non-Hispanic Black (OR 2.70, 95% CI 2.63–2.77), Hispanic/Latino (OR 2.45, 95% CI 2.38–2.51), and Other/Multiracial (OR 2.32, 95% CI 2.25–2.40) respondents, but were lower among Asian respondents (OR 0.75, 95% CI 0.71–0.79), controlling for other covariates. In aggregate, food insecurity was highest in ages <55; however, differentiation by sex as well as by race/ethnic group showed clear differences (Figure 4). The most evenly distributed food security status was for those aged 75 and above. Non-Hispanic White and Asian respondents had low food insecurity across all age groups, particularly among older adult groups, for both men and women. Meanwhile, non-Hispanic Black, Hispanic, and Other/Multiracial respondents consistently showed higher food insecurity among both men and women from ages 25–64. The region of the US had some significant impact on food security status, with respondents residing in southern states experiencing roughly 12% higher odds of food insecurity relative to northeastern states (95% CI 1.08–1.15).

As the most comprehensive model, Model 3 also included an evaluation of education level, income level, marital status, household structure, housing tenure, and employment status (Table 3). Holding other covariates constant, respondents were most likely to report being food-insecure if they had less than a Bachelor’s degree, had an income below USD 35,000, were not currently married, did not own their own home, or were not currently working for reasons of either involuntary unemployment or health-related or unknown reasons. Retired persons had a lower food insecurity risk (OR 0.75, 95% 0.71–0.78), as did households with either no children under 18 years old, or single adult households with at least one child. After controlling for these additional variables, however, we found the risk of food insecurity was attenuated for younger age groups as well as race/ethnic minority groups, and women became slightly more food-secure relative to men.

## 4. Discussion

Our findings that approximately 9.3% of US households sometimes or often experienced food insufficiency within the prior 7 days aligns with the values of 8.1–10.0% found by a study by Nagata et al. using early HPS data [29]. Additionally, we found food insecurity among older adults aged ≥65 was less than 5%, with at least 59% with lower odds of food insufficiency compared to respondents aged 45–54 years (adjusted OR: 0.41 or below, *p* < 0.001) indicating a significantly reduced risk (Table 3). Though counter to expectations regarding a potentially vulnerable population group, this is in agreement with findings from other studies. Early investigations into the impact of age on food security status during the COVID-19 pandemic indicated that older adults did not face a heightened risk of food insecurity compared to younger adults in the initial stages of the pandemic [17,30], and that those aged ≥55 were among the lowest at risk [18]. This remained the case across socioeconomic categories, in which Fang et al. found that in low-income households (<200% poverty level) age was negatively associated with food insecurity during the early months of the pandemic [19]. Our efforts therefore sought to characterize the differential impact of the pandemic on food insecurity among the different age groups, particularly older adults, over a prolonged period of data collection.

In our assessment of trends over time, we found a sharp rise in food insecurity prevalence that remained above baseline within the first year of the pandemic (Figure 2). The analysis of food insecurity trends over time also yielded an interesting differentiation between younger and older age groups. Food insecurity among younger adults (aged 25–54 years) peaked early within Period 1 of the pandemic, then had a marked decline at the start of Period 2, before gradually increasing again into Period 3, although without reaching the same level as in the initial months of the pandemic (Figure 3). Meanwhile, older adults (aged ≥65) experienced a less dramatic increase in food insecurity during Period 1, and only a slight shift back down in Period 2. The highest food insecurity proportion for this age group was reached in Period 3, the later stages of the pandemic. Indeed, the proportion of older adults reporting food insecurity increased by over 50% from Period 1 to Period 3 (Table 2).

A 2023 systematic review of 20 primarily cross-sectional studies of food insecurity in older adults in the United States between 2005 and 2019 concluded that the factors most consistently associated with food insecurity in older adults were those of established social determinants of health, such as race, gender, education, and income [31]. In our logistic regression models, after controlling for time period, increasingly lower odds of food insecurity were found among higher age groups. The relationship between food insecurity and socioeconomic condition is well established, and we similarly found that household income was strongly associated with food security status. Our model showed that lower income and education level, not owning one’s home, and any form of unemployment other than retirement were associated with higher odds of food insecurity. We also showed that nearly half (46%) of food-insecure respondents were renting their homes, indicating competition between paying rent and paying for food. Other researchers have found that, due to widespread economic impacts and the establishment of social distancing recommendations, the pandemic exacerbated pre-existing food insecurity among low-income, food-insecure households that already had difficulty managing expenses and household needs [6]. We found disparities in food insecurity present across certain population demographics, as Black, Hispanic/Latino, and Other/Multiracial respondents had 31–61% higher odds of food insecurity, controlling for other covariates. Other studies that focused on race/ethnic group differences in food insecurity have consistently found a similarly elevated prevalence among minority-led households, keeping other factors constant [5,7].

It is widely acknowledged that the initial disruptions to food production systems with the onset of the COVID-19 pandemic, alongside related spikes in food prices [2], particularly when further combined with job loss or other causes of financial hardship due to the pandemic [3,4,32], all impacted the ability to access and purchase food. Federal and state governmental efforts to offset these conditions included raised spending on USDA’s Supplemental Nutrition and Assistance Program and instituting temporary emergency allotments [33], and bolstering social services through nonprofit and public organizations [34]. The USDA Economic Research Service (ERS) estimates that 12% of the US population used SNAP per month on average in 2020 [33], and we similarly found 11.1% (range 5.8% to 13.9% by age group) of HPS respondents overall reported using SNAP. However, SNAP use was present at two to three times the proportion among food-insecure respondents compared to the food secure, ranging from 26.1% to 35.7% for food-insecure respondents, compared to 5.5% to 12.7% for those who were food-secure, depending on age group. These values show that SNAP was utilized by at least one quarter of older adults ≥65, and by approximately one third of adults under 65 years who were food-insecure. However, it is important to note that questions regarding SNAP use had among the highest rates of missingness (approximately 20%) of variables we evaluated, and that SNAP use was not captured consistently across all survey weeks studied; hence, the data was included only from August 2020 to May 2023.

Social support may also have been influential, such as the gathering of families together to attend to needs and reduce the isolation of more vulnerable individuals, including facilitating more consistent food procurement [35]. Food-insecure households tended to have more household members, but it is unclear if this was the case prior to the pandemic or occurred as a result. Further research may explore how changes in household structure affected food insecurity risk.

### Strengths and Limitations

Our analysis utilized the HPS because its coverage allowed for assessment of household food security and age in relation to a wide array of potentially influential factors throughout three years of data collected during the COVID-19 pandemic. There have been few other studies focused on older age, as most age-related studies were in relation to households with children. To our knowledge, there are no studies to date examining food insecurity over the course of the pandemic, and few beyond the early stage of the pandemic. As this survey question remained consistently reported over time, merging the records across biweekly surveys to evaluate the 3-year study period in aggregate provided important insight into changes over time.

The HPS’s question on food sufficiency of the prior 7 days is a validated measure intended to record rapid shifts in food security status. However, food insecurity can have many definitions, making it challenging to consistently ascertain and precisely interpret [24]. The 2021 ERS Food Security Supplement report estimated the national prevalence of household food insecurity at 10.2%, which is slightly higher than our value of 9.3% [36]. This likely stems from the difference in scope and timeframe of our respective measures, since the HPS’s food insufficiency question assesses the prior 7 days, while the full set of questions in the Household Food Security Survey Module (HFSSM) used for the ERS report represents household food access and intake for one calendar year [24,25]. Meanwhile, a wide-scale analysis of food insecurity prevalence in the first year of the COVID-19 pandemic was conducted by Niles et al., using a USDA six-item module across 18 study sites in 15 states and nationally. They found food insecurity prevalence ranged from 10.8% up to 68.7%, depending on state, and whether the respondents were from a convenience, representative, or high-risk sample [5], while the population of this study was designed to be nationally representative. Furthermore, this analysis did not extend to including lack of palatability (which represented 25% of responses) in defining food insecurity. When the definition of food insecurity to include undesirable changes to types of food was expanded in some other HPS-based studies [37,38], increases in levels of food insecurity among adults aged ≥60 years mirrored that of younger adults in the HPS [37]. Those findings underscore the complexities of food insecurity among older adults, as unpalatable foods or difficulty in eating certain foods (e.g., due to dentition) can lead to restricted dietary intake and malnutrition [39]. This variability in food insecurity definition, measure, and application presents challenges in generalizing the interpretation of findings and making comparisons across studies.

Finally, HPS methods to identify and select participants required a residential address registered with the Census Bureau that could be paired with an active email and/or mobile telephone number. This likely restricted participation of individuals with inconsistent housing or communication setup, and HPS collection methods using online surveys was predicted to have a lower response rate compared to the traditional mail type. Ultimately, the response rate for the survey weeks was only roughly 10%. Nonetheless, HPS’s form of implementation allowed for a balance of confidentiality protections, efficiency, cost, and timeliness of responses [22,40].

The pandemic revealed the national vulnerabilities related to food security and calls for national preparedness strategies, including systematic nutrition status monitoring as part of critical information collection and analysis.

## 5. Conclusions

Food insecurity during the COVID-19 pandemic varied across age groups, with a lower overall prevalence among older adults. Additional factors associated with food insecurity included racial/ethnic group, education level, household income, marital status and household structure, employment status, and household tenure. Controlling for these covariates was found to attenuate the risk of food insecurity among younger age groups and race/ethnic minority groups. However, trends and associated factors differed over time, underscoring the need for continuous monitoring and tailored interventions to mitigate food insecurity during prolonged emergencies. Our findings can assist public health agencies in managing food assistance resources nationally as the nation recovers from the COVID-19 pandemic. With this information, public health professionals can further direct needs assessments of these vulnerable groups in preparation for future crises. We also observed that questions related to income, housing tenure, food spending, and social assistance usage had the lowest response rates. This finding is essential for recognizing the challenges of establishing national representative samples, particularly during health crises. To leverage the national survey data and inform public health intervention strategies, organizations should augment efforts to better capture sensitive information, particularly from participants within vulnerable population groups.

## Figures and Tables

**Table 1:** Population characteristics by food security status, April 2020–May 2023.

Characteristic	Categories	Total	Food Secure	Food Insecure	*p*-Value
**All**	-	-	80.3%	9.3%	-
**Age group (years)**	25–34	20.0%	18.9%	24.9%	<0.001
	35–44	19.5%	18.6%	25.6%	
	45–54	17.9%	17.4%	21.7%	
	55–64	18.9%	19.4%	16.9%	
	65–74	16.8%	18.3%	8.3%	
	≥75	6.9%	7.5%	2.6%	
**Sex**	Male	48.0%	48.3%	45.1%	<0.001
	Female	52.0%	51.7%	54.9%	
**Race/Ethnicity**	Non-Hispanic White	63.0%	66.7%	44.3%	<0.001
	Non-Hispanic Black	11.7%	10.1%	20.5%	
	Hispanic/Latino	16.4%	14.4%	26.3%	
	Asian	5.1%	5.3%	2.9%	
	Other/Multiple	3.8%	3.5%	6.0%	
**Marital status**	Married	58.8%	61.8%	38.8%	<0.001
	Widowed/Divorced/Separated	19.9%	18.8%	29.2%	
	Never married	20.5%	19.0%	31.4%	
**Education**	High school graduate or less	38.4%	34.5%	59.6%	<0.001
	Some college/Associate’s degree	29.0%	29.1%	29.9%	
	Bachelor’s degree	17.8%	19.6%	6.7%	
	Graduate degree	14.8%	16.8%	3.8%	
**Income level**	<USD 35 K	19.3%	18.2%	49.5%	<0.001
	USD 35 K to < USD 75 K	23.3%	26.2%	24.1%	
	USD 75 K to < USD 150 K	22.3%	26.9%	7.2%	
	≥USD 150 K	11.7%	14.4%	1.3%	
**Employment status**	Currently working	55.8%	58.9%	42.4%	<0.001
	Retired	17.9%	20.0%	6.9%	
	Not working: involuntarily unemployed	6.7%	5.8%	16.3%	
	Not working: illness or caregiver role	7.5%	6.4%	17.4%	
	Not working: other or unknown reason	10.2%	8.6%	16.5%	
**Household structure**	1 adult, no children <18 yo	8.7%	8.8%	9.3%	<0.001
	2+ adults, no children <18 yo	52.7%	54.9%	40.3%	
	1 adult & children <18 yo	2.9%	2.5%	5.3%	
	2+ adults & children <18 yo	35.7%	33.8%	45.1%	
**Housing tenure**	Owned	19.8%	23.2%	11.7%	<0.001
	Mortgage or loan	37.3%	43.5%	24.3%	
	Rented	22.3%	22.4%	46.1%	
	Occupied without pay	1.3%	1.2%	4.1%	
**Region**	Northeast	17.4%	17.4%	15.9%	<0.001
	Midwest	20.6%	21.0%	18.5%	
	South	38.4%	37.6%	43.2%	
	West	23.6%	24.0%	22.4%	

Second order Rao-Scott chi-square test of significance.

**Table 2:** Proportion food insecure by population characteristics by time period.

Characteristic	Categories	% FI Period 1	% FI Period 2	% FI Period 3	Change >10%
**Age group (years)**	25–34	27.3%	23.2%	21.7%	− *
	35–44	26.1%	26.6%	23.5%	− *
	45–54	21.2%	21.9%	22.6%	+
	55–64	16.3%	16.7%	18.4%	+ *
	65–74	7.1%	8.5%	10.7%	+ *
	75+	2.1%	3.0%	3.2%	+ *
**Sex**	Male	45.6%	45.4%	43.5%	−
	Female	54.4%	54.6%	56.5%	+
**Race/Ethnicity**	Non-Hispanic White	43.3%	44.0%	46.9%	+
	Non-Hispanic Black	21.4%	20.0%	19.4%	−
	Hispanic/Latino	26.6%	27.2%	24.6%	−
	Asian	3.0%	2.8%	2.7%	−
	Other/Multiple	5.7%	6.0%	6.3%	+ *
**Marital status**	Married	38.4%	38.3%	40.1%	+
	Widowed/Divorced/Separated	28.6%	29.4%	30.2%	+
	Never married	32.4%	31.6%	29.2%	−
**Education**	High school graduate or less	60.1%	59.5%	58.6%	−
	Some college/Associate’s degree	29.6%	29.9%	30.6%	+
	Bachelor’s degree	6.8%	6.6%	6.9%	+
	Graduate degree	3.6%	4.0%	4.0%	+ *
**Income level**	< USD 35 K	50.0%	50.8%	46.8%	−
	USD 35 K to < USD 75 K	24.8%	22.0%	25.4%	+
	USD 75 K to < USD 150 K	7.6%	5.8%	8.0%	+
	≥ USD 150 K	1.2%	1.3%	1.5%	+ *
**Employment status**	Currently working	39.5%	43.4%	47.6%	+ *
	Retired	5.3%	7.8%	9.5%	+ *
	Not working: involuntarily unemployed	23.3%	11.6%	6.6%	− *
	Not working: illness or caregiver role	17.2%	17.4%	17.9%	+
	Not working: other or unknown reason	14.5%	19.1%	17.5%	+ *
**Household structure**	1 adult, no children <18 yo	8.5%	9.6%	10.4%	+ *
	2+ adults, no children <18 yo	39.0%	41.2%	42.2%	+
	1 adult & child/ren <18 yo	5.4%	5.2%	5.1%	−
	2+ adults & child/ren <18 yo	47.0%	44.0%	42.3%	− *
**Housing tenure**	Owned	10.6%	12.2%	13.7%	+ *
	Mortgage or loan	25.0%	22.8%	24.8%	−
	Rented	47.5%	45.8%	43.4%	−
	Occupied without pay	4.1%	4.4%	3.6%	− *
**Region**	Northeast	15.7%	15.8%	16.2%	+
	Midwest	18.4%	17.8%	19.4%	+
	South	43.2%	44.1%	42.2%	−
	West	22.7%	22.3%	22.1%	−

* Indicates the change (+ or −) in proportion of category respondents of those food-insecure from Period 1 to Period 3 is greater than 10% of the Period 1 proportion.

**Table 3:** Association of age group with odds of food insecurity adjusted for covariates.

Models	Covariates	Beta	OR	95% CI Lower OR	95% CI Upper OR
**MODEL 1**	(Intercept)	−1.94	0.14	0.14	0.15
**Age group** *(Ref: 45–54)*	25–34	0.16	**1.17**	1.13	1.21
	35–44	0.15	**1.17**	1.14	1.20
	55–64	−0.38	**0.68**	0.66	0.71
	65–74	−1.13	**0.32**	0.31	0.34
	75+	−1.46	**0.23**	0.21	0.26
**Time period** *(Ref: Period 1)*	Period 2	−0.10	**0.91**	0.87	0.95
	Period 3	0.14	**1.15**	1.10	1.20
**Interaction terms** *(Ref: 45–54 & Period 1)*	25–34 * Period 2	−0.15	**0.86**	0.81	0.92
	35–44 * Period 2	−0.04	0.96	0.91	1.01
	55–64 * Period 2	0.00	1.00	0.93	1.07
	65–74 * Period 2	0.11	**1.12**	1.05	1.19
	75+ * Period 2	0.26	**1.29**	1.11	1.51
	25–34 * Period 3	−0.32	**0.73**	0.69	0.77
	35–44 * Period 3	−0.20	**0.82**	0.78	0.86
	55–64 * Period 3	0.08	**1.08**	1.01	1.15
	65–74 * Period 3	0.29	**1.34**	1.24	1.46
	75+ * Period 3	0.35	**1.42**	1.22	1.67
**MODEL 2**	(Intercept)	−2.41	0.09	0.09	0.09
**Age group** *(Ref: 45–54)*	25–34	0.13	**1.14**	1.10	1.18
	35–44	0.12	**1.13**	1.10	1.16
	55–64	−0.34	**0.71**	0.68	0.74
	65–74	−1.01	**0.36**	0.34	0.38
	75+	−1.28	**0.28**	0.25	0.31
**Time period** *(Ref: Period 1)*	Period 2	−0.11	**0.90**	0.86	0.94
	Period 3	0.13	**1.14**	1.09	1.19
**Sex** *(Ref: Male)*	Female	0.12	**1.13**	1.11	1.15
**Race/Ethnicity** *(Ref: non-Hispanic White)*	Non-Hispanic Black	0.99	**2.70**	2.63	2.77
	Hispanic/Latino	0.89	**2.45**	2.38	2.51
	Asian	−0.29	**0.75**	0.71	0.79
	Other/Multiracial	0.84	**2.32**	2.25	2.40
**Region**	Midwest	0.05	**1.05**	1.02	1.08
	South	0.11	**1.12**	1.08	1.15
	West	−0.07	**0.93**	0.90	0.96
**Interaction terms** *(Ref: 45–54 & Period 1)*	25–34 * Period 2	−0.11	**0.89**	0.84	0.95
	35–44 * Period 2	−0.02	0.98	0.93	1.03
	55–64 * Period 2	0.01	1.01	0.94	1.08
	65–74 * Period 2	0.11	**1.12**	1.04	1.19
	75+ * Period 2	0.24	**1.27**	1.09	1.48
	25–34 * Period 3	−0.28	**0.76**	0.71	0.80
	35–44 * Period 3	−0.18	**0.83**	0.79	0.88
	55–64 * Period 3	0.07	**1.08**	1.01	1.15
	65–74 * Period 3	0.30	**1.35**	1.25	1.47
	75+ * Period 3	0.34	**1.41**	1.20	1.66
**MODEL 3**	(Intercept)	−3.18	0.04	0.04	0.04
**Age group** *(Ref: 45–54)*	25–34	−0.11	**0.90**	0.86	0.94
	35–44	0.07	**1.07**	1.03	1.12
	55–64	−0.35	**0.70**	0.67	0.73
	65–74	−0.89	**0.41**	0.38	0.44
	75+	−1.23	**0.29**	0.26	0.33
**Time period** *(Ref: Period 1)*	Period 2	−0.03	0.97	0.92	1.02
	Period 3	0.33	**1.39**	1.31	1.47
**Sex** *(Ref: Male)*	Female	−0.09	**0.92**	0.90	0.94
**Race/Ethnicity** *(Ref: non-Hispanic White)*	Non-Hispanic Black	0.33	**1.40**	1.35	1.44
	Hispanic/Latino	0.27	**1.31**	1.27	1.35
	Asian	−0.15	**0.86**	0.80	0.92
	Other/Multiracial	0.48	**1.62**	1.56	1.67
**Region**	Midwest	−0.01	0.99	0.96	1.03
	South	0.08	**1.08**	1.04	1.11
	West	−0.11	**0.90**	0.86	0.93
**Education** *(Ref: Bachelor’s degree)*	High school graduate or less	0.82	**2.28**	2.21	2.35
	Some college/Associate’s degree	0.61	**1.84**	1.78	1.89
	Graduate degree	−0.09	**0.91**	0.88	0.95
**Income group** *(Ref: USD 35 K to < USD 75 K)*	< USD 35 K	0.71	**2.03**	1.98	2.08
	USD 75 K to < USD 150 K	−0.94	**0.39**	0.37	0.41
	≥ USD 150 K	−1.73	**0.18**	0.16	0.19
**Marital status** *(Ref: Married)*	Widowed/Divorced/Separated	0.41	**1.50**	1.45	1.55
	Never married	0.20	**1.22**	1.18	1.26
**Household structure** *(Ref: 2+ adults, children)*	1 adult, no children	−0.38	**0.68**	0.66	0.71
	2+ adults, no children <18 yo	−0.19	**0.83**	0.81	0.85
	1 adult & child/children <18 yo	−0.19	**0.82**	0.79	0.86
**Housing tenure** *(Ref: Owned)*	Mortgage or loan	0.18	**1.19**	1.16	1.23
	Rented	0.63	**1.87**	1.81	1.93
	Occupied without payment	1.05	**2.85**	2.68	3.04
**Employment status** *(Ref: currently working)*	Not working: retired	−0.29	**0.75**	0.71	0.78
	Not working: involuntarily unemployed	0.86	**2.37**	2.27	2.47
	Not working: personal illness/caregiver	0.59	**1.81**	1.74	1.88
	Not working: other/unknown reason	0.45	**1.57**	1.52	1.62
**Interaction terms** *(Ref: 45–54 & Period 1)*	25–34 * Period 2	−0.09	**0.91**	0.84	0.99
	35–44 * Period 2	−0.04	0.96	0.90	1.03
	55–64 * Period 2	−0.01	0.99	0.92	1.07
	65–74 * Period 2	0.13	**1.14**	1.05	1.24
	75+ * Period 2	0.14	1.15	0.97	1.36
	25–34 * Period 3	−0.21	**0.81**	0.75	0.88
	35–44 * Period 3	−0.16	**0.85**	0.80	0.92
	55–64 * Period 3	−0.04	**0.97**	0.89	1.05
	65–74 * Period 3	0.17	**1.19**	1.08	1.31
	75+ * Period 3	0.19	**1.21**	1.01	1.45

Interaction designated with * in between terms.

## Data Availability

Dataset is publicly available: https://www.census.gov/programs-surveys/household-pulse-survey/datasets.html (accessed on 19 October 2022).

## Supplementary Materials

The following supporting information can be downloaded at: https://www.mdpi.com/article/10.3390/ijerph21081078/s1, Table S1: Food program use and food spending in total and by food security status; Table S2: Food security status by population characteristic for males and females.
