# Supplementary material for: Food Insecurity across Age Groups in the United States during the COVID-19 Pandemic
Source: Int J Environ Res Public Health. 2024 Aug 16;21(8):1078. doi: 10.3390/ijerph21081078 (PMC11353888; doi:10.3390/ijerph21081078)
Supplement: Supplementary file 1 [file ijerph-21-01078-s001.zip › ijerph-3076616-supplementary.pdf]

Supplemental Table S1. Food program use and food spending in total and by food security status

Survey Weeks	Support	Response	Total	Food Secure	Food Insecure	p-value
Weeks 13-57	**SNAP use**	**No**	70.00%	81.90%	60.50%	<0.001
		**Yes**	11.10%	10.30%	32.10%	
Weeks 1-57	**Received free groceries or meals**	**No**	82.30%	92.90%	78.60%	<0.001
		**Yes**	6.80%	6.20%	19.10%	
Survey Weeks	**Spending**	**Category**	**Mean**	**Mean**	**Mean**	**p-value**
1-34, 46-57	**Money spent on food in last week per household ($)**	**Food & Groceries**	224.1	222.1	238.9	<0.001
		**Prepared meals**	89.6	89	93.9	<0.001
		**Category**	**Median (IQR)**	**Median (IQR)**	**Median (IQR)**	**p-value**
		**Food & Groceries**	200 (100-300)	200 (100-300)	200 (100-300)	n/a
		**Prepared meals**	50 (20-100)	50 (20-100)	40 (0-120)	n/a
	**Money spent on food in last week per capita ($)**	**Category**	**Median (IQR)**	**Median (IQR)**	**Median (IQR)**	**p-value**
		**Food & Groceries**	65 (40-100)	66.7 (42-100)	50 (26.7-100)	n/a
		**Prepared meals**	20 (6-40)	20 (6.7-40)	10 (0-37.5)	n/a

Supplemental Table S2. Food security status by population characteristic for males and females

Characteristic	Sex	Categories	Missing	Food Secure	Food Insecure	Total
**Age Group**	Male	25-34	0.24	0.19	0.25	0.20
		35-44	0.22	0.19	0.25	0.20
		45-54	0.19	0.18	0.21	0.18
		55-64	0.18	0.20	0.17	0.19
		65-74	0.12	0.17	0.08	0.16
		75+	0.06	0.08	0.03	0.07
	Female	25-34	0.24	0.19	0.25	0.20
		35-44	0.21	0.18	0.26	0.19
		45-54	0.18	0.17	0.22	0.17
		55-64	0.17	0.19	0.17	0.19
		65-74	0.15	0.20	0.09	0.18

		75+	0.06	0.07	0.02	0.07
**Race/ Ethnicity**	Male	Non-Hispanic White	0.52	0.67	0.44	0.64
		Non-Hispanic Black	0.15	0.09	0.18	0.11
		Hispanic/Latino	0.23	0.15	0.28	0.17
		Asian	0.06	0.06	0.04	0.06
		Other/Multiple	0.04	0.03	0.05	0.03
	Female	Non-Hispanic White	0.51	0.66	0.44	0.62
		Non-Hispanic Black	0.17	0.11	0.22	0.13
		Hispanic/Latino	0.23	0.14	0.25	0.16
		Asian	0.05	0.05	0.02	0.05
		Other/Multiple	0.04	0.04	0.06	0.04
**Marital Status**	Male	Missing	0.05	0.00	0.01	0.01
		Married	0.57	0.66	0.43	0.63
		Widowed/Divorced/Separated	0.15	0.13	0.23	0.14
		Never married	0.23	0.20	0.33	0.21
	Female	Missing	0.05	0.00	0.01	0.01
		Married	0.50	0.57	0.35	0.54
		Widowed/Divorced/Separated	0.24	0.24	0.34	0.25
		Never married	0.22	0.18	0.30	0.20
**Education**	Male	High School graduate or less	0.53	0.36	0.62	0.40
		Some college/Associate's degree	0.26	0.28	0.27	0.28
		Bachelor's degree	0.13	0.19	0.07	0.17
		Graduate degree	0.09	0.16	0.04	0.15
	Female	High School graduate or less	0.47	0.33	0.57	0.37
		Some college/Associate's degree	0.29	0.30	0.32	0.30
		Bachelor's degree	0.14	0.20	0.07	0.18
		Graduate degree	0.10	0.17	0.04	0.15
**Income Level**	Male	Missing	0.98	0.14	0.19	0.23
		< $35K	0.01	0.15	0.44	0.16
		$35K to < $75K	0.01	0.26	0.26	0.23
		$75K to <$150K	0.00	0.29	0.09	0.24
		$150K+	0.00	0.17	0.02	0.14
	Female	Missing	0.98	0.15	0.17	0.24
		< $35K	0.01	0.21	0.54	0.22
		$35K to < $75K	0.01	0.27	0.23	0.24
		$75K to <$150K	0.00	0.25	0.06	0.21
		$150K+	0.00	0.12	0.01	0.10
**Employment Status**	Male	Missing	0.16	0.00	0.01	0.02
		Currently working	0.47	0.64	0.43	0.60
		Retired	0.10	0.19	0.07	0.17
		Not working, involuntarily unemployed	0.06	0.06	0.20	0.07

		Not working, personal illness or caregiver role	0.05	0.04	0.13	0.05
		Not working, other or unknown reason	0.16	0.07	0.16	0.09
	Female	Missing	0.16	0.00	0.00	0.02
		Currently working	0.40	0.54	0.42	0.52
		Retired	0.12	0.21	0.07	0.19
		Not working, involuntarily unemployed	0.05	0.06	0.14	0.06
		Not working, personal illness or caregiver role	0.09	0.09	0.21	0.10
		Not working, other or unknown reason	0.18	0.10	0.17	0.11
**Household Structure**	Male	1 adult, no children	0.07	0.08	0.11	0.08
		2+ adults, no children <18 yo	0.49	0.57	0.45	0.55
		1 adult & child/children <18 yo	0.03	0.02	0.03	0.02
		2+ adults & child/children <18 yo	0.41	0.33	0.41	0.34
	Female	1 adult, no children	0.07	0.09	0.08	0.09
		2+ adults, no children <18 yo	0.44	0.53	0.37	0.50
		1 adult & child/children <18 yo	0.05	0.03	0.07	0.04
		2+ adults & child/children <18 yo	0.43	0.35	0.48	0.37
**Housing Tenure**	Male	Missing / Did not report	0.97	0.10	0.15	0.20
		Owned	0.01	0.23	0.12	0.20
		Mortgage or loan	0.01	0.44	0.26	0.38
		Rented	0.01	0.21	0.42	0.21
		Occupied without pay	0.00	0.01	0.05	0.01
	Female	Missing / Did not report	0.97	0.10	0.13	0.19
		Owned	0.01	0.23	0.11	0.20
		Mortgage or loan	0.01	0.43	0.23	0.36
		Rented	0.01	0.24	0.49	0.24
		Occupied without pay	0.00	0.01	0.03	0.01
**Region**	Male	Northeast	0.19	0.17	0.16	0.17
		Midwest	0.19	0.21	0.18	0.21
		South	0.40	0.37	0.42	0.38
		West	0.22	0.24	0.23	0.24
	Female	Northeast	0.19	0.18	0.16	0.18
		Midwest	0.19	0.21	0.19	0.20
		South	0.41	0.38	0.44	0.39
		West	0.21	0.24	0.22	0.23
**SNAP**	Male	Missing / Did not report	0.97	0.08	0.08	0.19
		Yes	0.01	0.08	0.26	0.08
		No	0.03	0.84	0.66	0.73
	Female	Missing / Did not report	0.96	0.08	0.07	0.19
		Yes	0.01	0.13	0.37	0.14
		No	0.02	0.80	0.56	0.68
**Free Food**	Male	Missing / Did not report	0.96	0.01	0.02	0.11

**Receipt**		Yes	0.00	0.05	0.18	0.06
		No	0.04	0.94	0.80	0.83
	Female	Missing / Did not report	0.96	0.01	0.02	0.11
		Yes	0.01	0.07	0.20	0.08
		No	0.03	0.92	0.78	0.81

* Each food security category was normalized within each sex and variable
